# Is it possible to use complete blood collection based systemic inflammatory indices as potential biomarkers for chronic spontaneous urticaria

**DOI:** 10.3389/fimmu.2026.1760879

**Published:** 2026-03-09

**Authors:** Bingyu Li, Lu Peng, Runqing Li, Mai Shi, Yingyi Li, Chinghsuan Sun, Zhuying Zhang, Jingwen Xue, Yi Zhao

**Affiliations:** 1 Department of Dermatology, Beijing Tsinghua Changgung Hospital, School of Clinical Medicine, Tsinghua Medicine, Tsinghua University, Beijing, China; 2 Photomedicine Laboratory, Institute of Precision Medicine, Tsinghua University, Beijing, China; 3 Department of Laboratory Medicine, Beijing Tsinghua Changgung Hospital, School of Clinical Medicine, Tsinghua Medicine, Tsinghua University, Beijing, China

**Keywords:** biomarkers, chronic spontaneous urticaria, inflammation, MPV, NLR, PDW, platelet, PLR

## Abstract

Chronic spontaneous urticaria (CSU) is mediated not only by mast cells but also by eosinophils and basophils. We evaluated whether complete blood collection based systemic inflammatory indices- including the systemic immune-inflammation index (SII), systemic inflammation response index (SIRI), aggregate Index of Systemic Inflammation (AISI), systemic inflammation modulation index (SIMI), neutrophil-to-lymphocyte ratio (NLR), eosinophil-to-lymphocyte ratio (ELR), platelet-to-lymphocyte ratio (PLR), basophil-to-lymphocyte ratio (BLR)-and platelet parameters (mean platelet volume [MPV], platelet distribution width [PDW], platelet large cell ratio [PLCR]) reflect CSU severity or treatment response. A retrospective study of 190 CSU patients and 570 matched controls was performed, with sensitivity analyses using propensity-score matching (PSM) and inverse-probability-of-treatment weighting (IPTW). Subgroup analyses examined UAS7, antihistamine response and allergy history. As a result, CSU patients exhibited lower SII/SIRI, white blood cells (WBC), neutrophils, lymphocytes, and NLR, alongside higher MPV/PLCR and reduced PDW. NLR showed a weak correlation with UAS7, and systemic indices did not reliably differentiate standard-dosed and updosed antihistamine response. Patients with allergy history demonstrated lower eosinophils and ELR. CSU is characterized by reduced systemic inflammatory indices and enhanced platelet activation. Among these, NLR may serve as a cost-effective supplementary tool for assessing systemic inflammation trends of CSU.

## Introduction

Chronic spontaneous urticaria (CSU) is a mast cell–mediated disorder characterized by recurrent wheals, angioedema, or both, for a minimum of six weeks in the absence of identifiable exogenous triggers (1). Guiding management of CSU according to treatment goals involves serial patient-reported monitoring with the Urticaria Activity Score 7 (UAS7) and Urticaria Control Test (UCT) (2). However, the subjective nature of both UAS7 and UCT highlights a need for cost-effective, objective biomarkers to facilitate more reliable disease assessment in routine clinical practice (3).

Emerging evidence indicates that systemic inflammation significantly contributes to the pathogenesis of CSU, driven by mediators such as tumor necrosis factor-α, interleukin-17, other cytokines, chemokines, and serum autoantibodies (4). Cost-effective, complete blood collection based systemic inflammation biomarkers are increasingly recognized as hallmarks of the systemic inflammatory response in immune-mediated allergic diseases or skin disorders (5–7). Complete blood collection-based systemic inflammation biomarkers include systemic immune-inflammation index (SII), systemic inflammation response index (SIRI), neutrophil−to−lymphocyte ratio (NLR), eosinophil−to−lymphocyte ratio (ELR), platelet−to−lymphocyte ratio (PLR), basophil−to−lymphocyte ratio (BLR); platelet parameters including mean platelet volume (MPV), platelet distribution width (PDW), platelet large cell ratio (PLCR), aggregate index of systemic inflammation (AISI) and systemic inflammation modulation index (SIMI). These indices involve multiple leukocyte and platelet lineages, reflecting distinct pathways of systemic immune-inflammatory responses. In addition, these indices are readily obtained in routine clinical practice with minimal patient burden, rendering them attractive adjunctive tools.

A few studies have evaluated complete blood-based systemic inflammation biomarkers in patients with CSU, and their findings remain heterogeneous. A hospital-based retrospective study (143 CSU patients; 132 controls) found that CSU patients had higher MPV and lower PDW vales at baseline compared to healthy controls. During omalizumab therapy, MPV, MPV/platelet count, and PDW increased while NLR decreased, suggesting concurrent platelet activation and an anti-inflammatory effect (8). In contrast, a retrospective cohort of 52 CSU patients receiving omalizumab 300 mg every four weeks reported no significant longitudinal changes in NLR/ELR/PLR/BLR and limited predictive value for response, although baseline ratios correlated with CRP, total IgE, and thyroid autoimmunity (9). These discrepancies are likely attributable to variations in cohort composition, endotype mix, and the timing of sampling relative to disease flares or concomitant therapy. Therefore, more studies are needed to further determine the clinical utility and reliability of these inflammatory biomarkers in CSU management.

Here, we conducted a propensity score−matched comparison of complete blood collection based systemic immune-inflammation index between CSU and healthy controls, and evaluated their associations with disease severity and treatment response.

## Materials and methods

### Study design and participants

This retrospective, single-center study enrolled patients diagnosed with CSU who visited the Department of Dermatology at Beijing Tsinghua Changgung Hospital between March 2021 and April 2025. A total of 206 patients were initially screened. Sixteen individuals without baseline hematological examinations at the time of CSU diagnosis were excluded, resulting in 190 eligible patients for the final analysis. The diagnosis of CSU was established based on the 2022 EAACI/GA²LEN/EuroGuiDerm/APAAACI guideline, requiring recurrent wheals and/or angioedema persisting for more than six weeks without identifiable external triggers.

**Figure 1:**
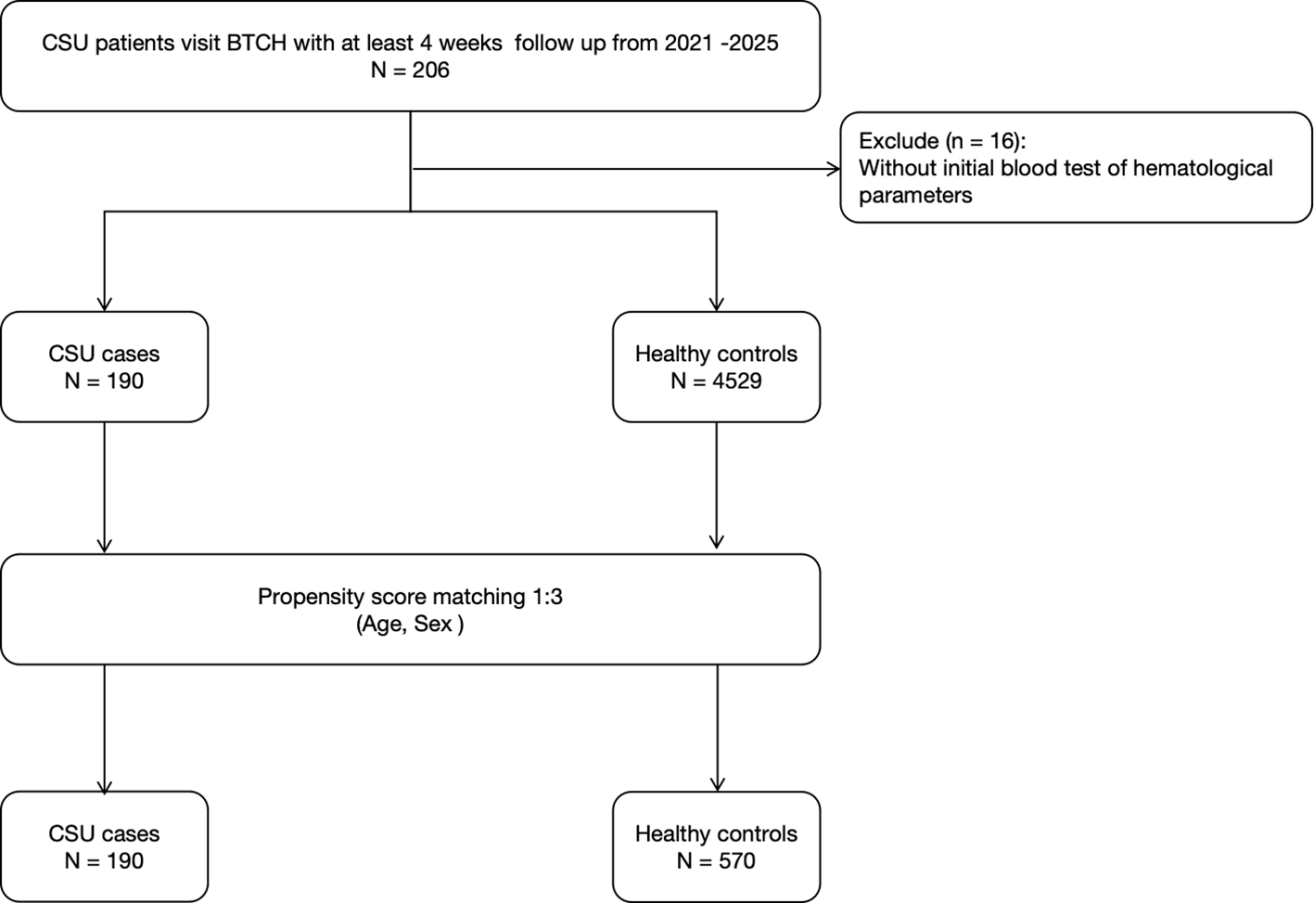
Flow diagram of patient inclusion, exclusion, and matching process.

A total of 4529 non-CSU controls were recruited from the hospital’s physical examination center during the same period. These controls had no personal or family history of urticaria, autoimmune diseases, or chronic inflammatory disorders. To minimize confounding effects of age and sex, propensity score matching (PSM) was applied at a 1:3 ratio with 190 CSU patients *vs*. 570 healthy controls. The overall study flow is presented in **Figure 1**, and the balance of covariates before and after matching was evaluated using standardized mean differences (SMD) displayed in **Supplementary Figure 1**.

### Data collection and laboratory measurements

Demographic and clinical characteristics, including age, sex, baseline CSU severity assessed by UAS7, treatment response and allergy history, were obtained from electronic medical records. Venous blood samples were collected at baseline prior to systemic or biological treatment. Hematological parameters were analyzed using an automated hematology analyzer (Sysmex XN series, Japan). The assessed indicators included WBC, neutrophil, lymphocyte, monocyte, eosinophil, basophil, platelet count (PLT), PDW, MPV, and PLCR. Based on these parameters, several composite inflammatory indices were calculated to comprehensively assess systemic inflammatory status. NLR, PLR, BLR and ELR were derived from corresponding leukocyte and platelet counts (10, 11). In addition SII was determined as the product of the platelet and neutrophil counts divided by the lymphocyte count, whereas SIRI was computed as the product of the neutrophil and monocyte counts divided by the lymphocyte count. AISI was calculated as the product of the neutrophil count, monocyte count, and platelet count divided by the lymphocyte count. SIMI was calculated as the monocyte count multiplied by the platelet count and divided by the lymphocyte count (12, 13).

### Statistical analysis

PSM was performed using a nearest-neighbor algorithm with ratio of 1:3, adjusting for age and sex. Balance after matching was assessed using SMD, and covariates were considered well-balanced when SMD < 0.1 (14, 15). For sensitivity analyses, two additional methods were applied: inverse probability of treatment weighting (IPTW) and PSM with ratio 1.

Continuous variables were expressed as mean ± standard deviation (SD), and categorical variables as counts and percentages. Between-group comparisons were conducted using the Wilcoxon rank-sum test. Spearman’s rank correlation was used to explore associations between hematological markers and disease activity scores. Statistical significance was set at *p* < 0.05. All analyses were performed in R (version 4.3.3; R Foundation for Statistical Computing, Vienna, Austria).

### Sensitivity and subgroup analyses

To further examine the robustness and internal consistency of the primary findings, a series of sensitivity and subgroup analyses were performed. Sensitivity analyses included two alternative propensity score–based approaches: IPTW and 1:1 nearest-neighbor PSM. Both methods demonstrated similar covariate balance to the primary 1:3 PSM analysis.

Subgroup analyses were then conducted to explore the associations of complete blood collection based systemic immune-inflammation index with disease severity and treatment response of CSU. First, correlations between continuous UAS7 scores and systemic immune-inflammation index were evaluated using Spearman’s rank correlation coefficients. Second, clinical response to standard-dosed and updosed(fourfold) antihistamine therapy were compared between responders and nonresponders after 1:1 matching with 0.2 caliper on the same covariates respectively. All these two PSM analyses were matched with age and gender. Finally, inflammatory profiles were compared between patients with and without a self-reported allergy history, applying 1:3 matching on age and sex. Matching ratios were determined according to sample size and balance diagnostics. Covariate balance was assessed using SMD, with SMD < 0.1 indicating adequate balance.

## Results

### Study population

A total of 190 patients with confirmed CSU with mean age, 41.90 ± 15.34 years; 65% female, and 570 age and sex-matched non-CSU controls with mean age, 41.14 ± 15.38 years; 66% female was included after 1:3 propensity score matching. Baseline demographic and hematological characteristics of both groups are summarized in **Table 1**.

**Table 1:** Clinicopathological parameters in CSU patients and healthy control subjects.

Variable	Controls^1^	Cases^1^	*P* value^2^
Sex			0.826
Female	374 (66%)	123 (65%)	
Male	196 (34%)	67 (35%)	
Age	41.16 ± 15.39	41.94 ± 15.39	0.699
WBC	6.68 ± 1.67	5.86 ± 1.47	<0.001*
Monocyte	0.40 ± 0.33	0.37 ± 0.11	0.972
Neutrophil	4.18 ± 1.75	3.35 ± 1.14	<0.001*
Lymphocyte	2.09 ± 0.55	1.97 ± 0.57	0.010*
Eosinophil	0.16 ± 0.18	0.14 ± 0.11	0.162
PCT	0.25 ± 0.05	0.25 ± 0.06	0.034*
Basophil	0.02 ± 0.03	0.02 ± 0.02	<0.001*
PLT	258.81 ± 60.46	245.41 ± 59.01	0.006*
PDW	15.79 ± 1.40	13.73 ± 2.40	<0.001*
MPV	9.54 ± 1.15	10.46 ± 1.01	<0.001*
PLCR	23.17 ± 7.93	28.83 ± 7.60	<0.001*
SII	565.07 ± 341.91	448.75 ± 239.10	<0.001*
SIRI	0.91 ± 1.12	0.69 ± 0.43	0.004*
NLR	2.16 ± 1.20	1.82 ± 0.83	<0.001*
ELR	0.08 ± 0.08	0.07 ± 0.06	0.811
BLR	0.01 ± 0.02	0.01 ± 0.01	<0.001*
PLR	131.96 ± 46.41	133.69 ± 48.07	0.580
AISI	238.14 ± 304.67	172.70 ± 123.87	<0.001*
SIMI	52.75 ± 57.66	48.79 ± 20.57	0.517

^1^n (%); Median (Q1, Q3).

^2^Pearson’s Chi-squared test; Wilcoxon rank sum test.

WBC, white blood cell; NEUT, neutrophil; LYM, lymphocyte; MONO, monocyte; EOS, eosinophil; BASO, basophil; PLT, platelet count; SII, the systemic immune-inflammation index; SIRI, systemic inflammation response index; NLR, neutrophil−to−lymphocyte ratio; ELR, eosinophil−to−lymphocyte ratio; PLR, platelet−to−lymphocyte ratio; BLR, basophil−to−lymphocyte ratio; MPV, platelet parameters including mean platelet volume; PDW, platelet distribution width; *: Statistically significant difference (*p* <.05).

### Comparison between CSU and non-CSU controls

**Figure 2:**
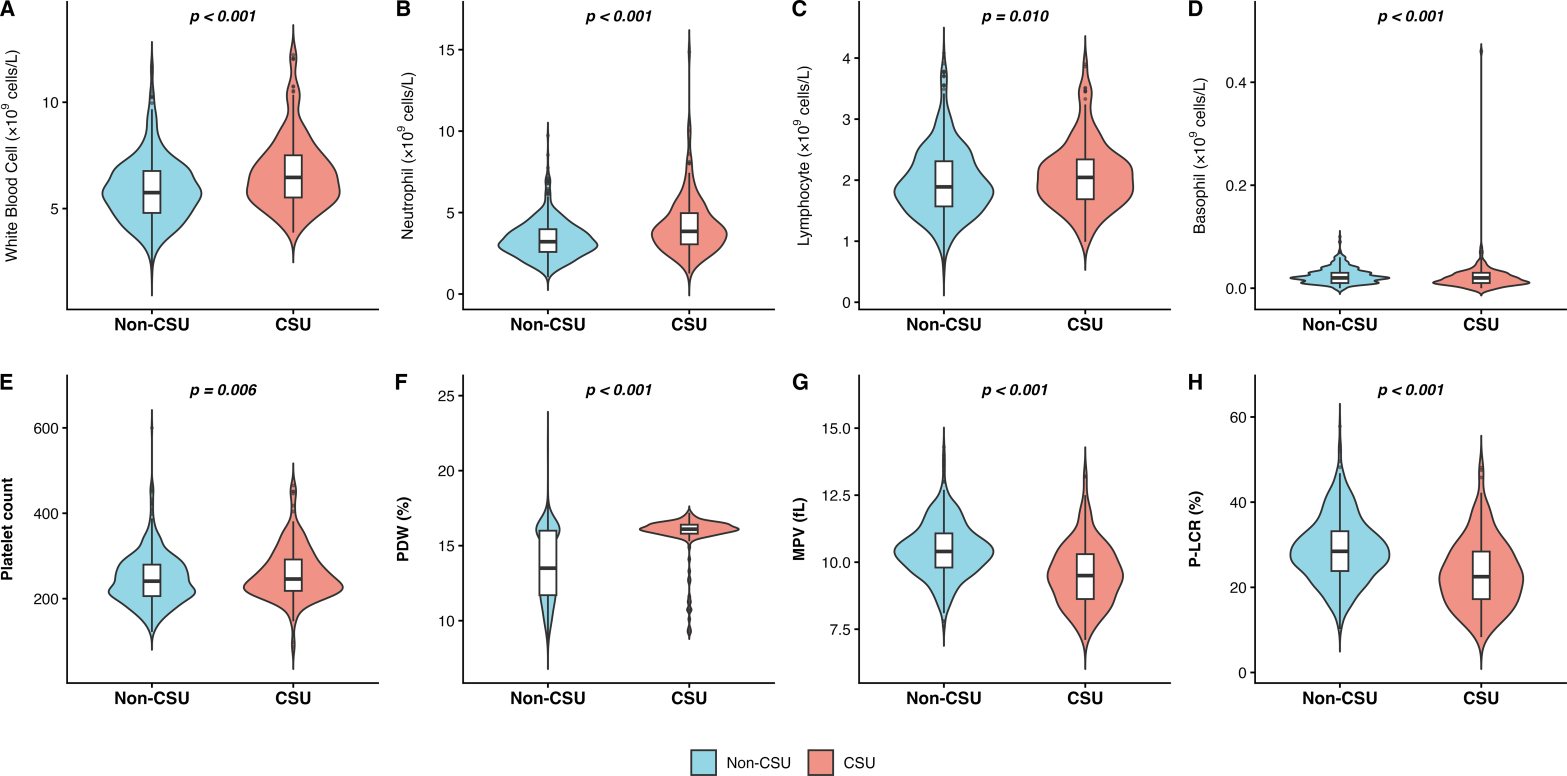
Eight violin plots comparing blood parameters between Non-CSU and CSU groups, showing statistically significant differences in white blood cell, neutrophil, lymphocyte, basophil counts, platelet count, PDW, MPV, and P-LCR, with Non-CSU in blue and CSU in red. Each is labeled A-H with corresponding p-values indicated above the plots. A color key below indicates group colors and labels. **(A)**: Comparison of white blood cell count between Non-CSU and CSU groups. **(B)**: Distribution of neutrophil count in Non-CSU versus CSU patients. **(C)**: Differential analysis of lymphocyte count across study groups. **(D)**: Significant elevation of basophil count in the CSU cohort. **(E)**: Comparative assessment of platelet count between groups. **(F)**: Distribution of platelet distribution width (PDW) in CSU and controls. **(G)**: Reduction of mean platelet volume (MPV) in CSU patients. **(H)**: Comparison of platelet-large cell ratio (P-LCR) between study populations.

**Figure 3:**
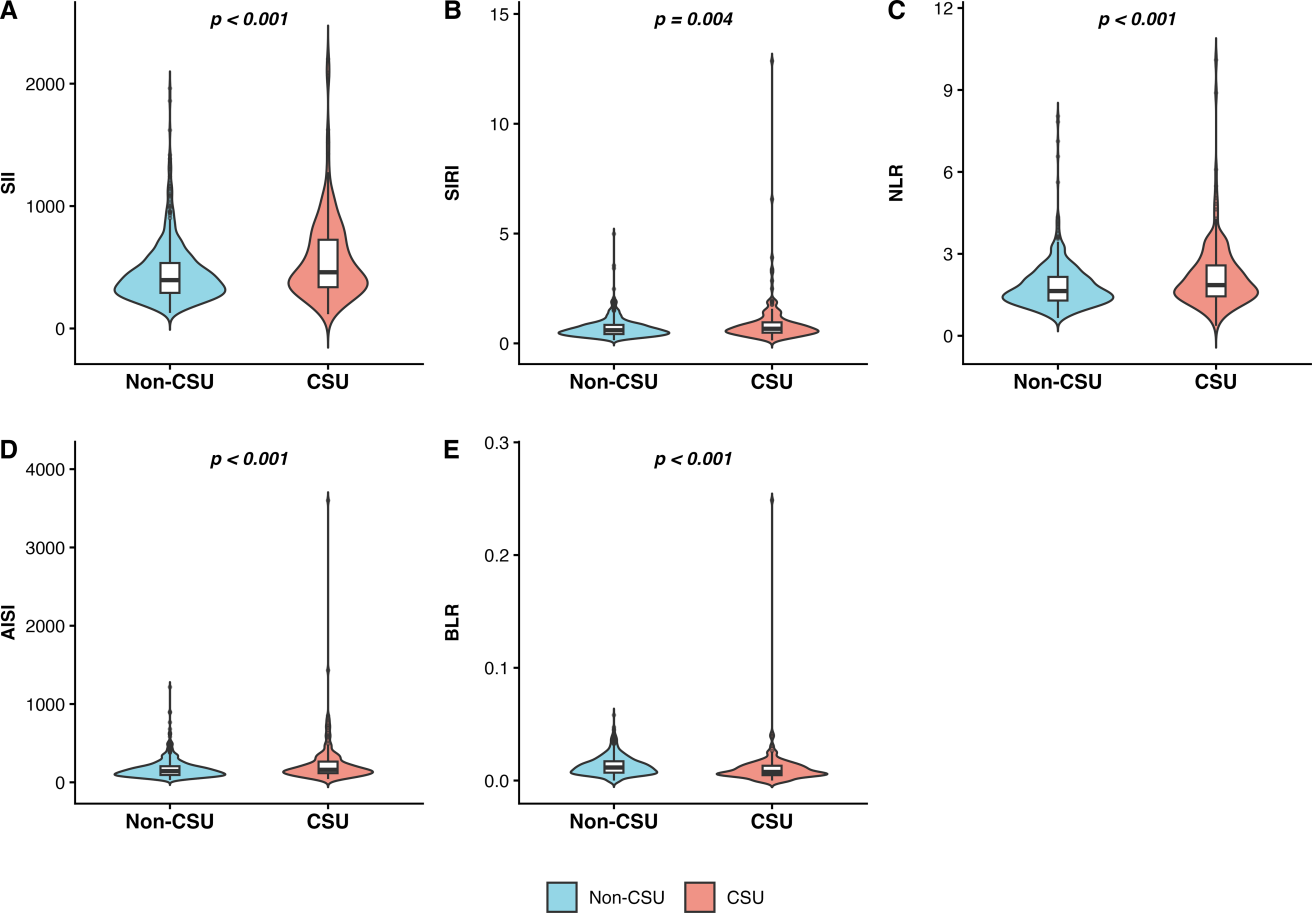
Violin plots compare five blood inflammation indices (SII, SIRI, NLR, BLR, AISI) between non-CSU (blue) and CSU (red) groups, with CSU values generally higher. P-values indicate statistically significant differences in all s. **(A)**: Comparison of Systemic Immune-Inflammation Index (SII) between Non-CSU and CSU groups. **(B)**: Distribution of Systemic Inflammation Response Index (SIRI) in Non-CSU versus CSU patients. **(C)**: Analysis of Neutrophil-to-Lymphocyte Ratio (NLR) across study cohorts. **(D)**: Comparison of Aggregate Index of Systemic Inflammation (AISI) between study groups. **(E)**: Significant differences in Basophil-to-Lymphocyte Ratio (BLR) between Non-CSU and CSU populations.

Several hematological and inflammatory parameters significantly differed between patients with CSU and non-CSU controls (**Table 1**). Compared with controls, CSU patients demonstrated lower total WBC counts (5.86 ± 1.47 *vs*. 6.68 ± 1.67 ×10^9^/L, *p* < 0.001; **Figure 2A**), lower neutrophil (3.35 ± 1.14 *vs*. 4.18 ± 1.75 ×10^9^/L, *p* < 0.001; **Figure 2B**) and LYM counts (1.97 ± 0.57 *vs*. 2.09 ± 0.55 ×10^9^/L, *p* = 0.010; **Figure 2C**). Conversely, BASO (0.02 ± 0.03 0.02 ± 0.02, *p* <0.001; **Figure 2D**) platelet-related parameters were significantly elevated in CSU patients, including MPV (10.46 ± 1.01 *vs*. 9.54 ± 1.15 fL, *p* < 0.001; **Figures 2E–G**) and PLCR (28.83 ± 7.60 *vs*. 23.17 ± 7.93, *p* < 0.001; **Figure 2H**). Furthermore, composite inflammatory indices, including SII (448.75 ± 239.10 *vs*. 565.07 ± 341.91, *p* < 0.001; **Figure 3A**), SIRI (0.69 ± 0.43 *vs*. 0.91 ± 1.12, *p* = 0.004), NLR (1.82 ± 0.83 *vs*. 2.16 ± 1.20, *p* < 0.001; **Figure 3B**) NLR (1.82 ± 0.83 vs. 2.16 ± 1.20, *p* < 0.001; **Figure 3C**) and AISI (172.70 ± 123.87 *vs*. 238.14 ± 304.67, p < 0.001; **Figure 3D**), were markedly decreased in the CSU group. In contrast, BLR was significantly lower among CSU patients (*p* < 0.001; **Figure 3E**), while no significant differences were observed for monocyte counts, eosinophil counts, ELR, PLR or SIMI(all *p* > 0.05).

### Sensitivity analyses

Two alternative methods, 1:1 PSM and IPTW, were conducted with SMD <0.1 (**Supplementary Figures 2**, **3**). Both models yielded consistent results with the main 1:3 PSM analysis, confirming that WBC, neutrophil and neutrophil-related and platelet- related indices (PDW, MPV, PLCR, SII, SIRI, NLR) remained significantly altered in CSU patients. The only discrepancy was observed for BLR, which lost significance under the IPTW approach, possibly due to weight-induced variance attenuation inherent in weighted estimations.

### Subgroup analyses

To further explore the clinical heterogeneity of CSU and verify the stability of the primary findings, a series of subgroup analyses were conducted across different clinical contexts from following three perspectives (**Tables 2**–**5**):

**Table 2:** Spearman correlation between hematologic markers and UAS7 score.

Biomarker	Spearman’s ρ	*P* value
MONO	-0.086	0.2440
NEUT	0.143	0.0517
PLT	0.013	0.8620
LYM	-0.109	0.1380
EOS	0.029	0.6940
BASO	-0.008	0.9090
PCT	0.066	0.3720
WBC	0.062	0.3980
PDW	0.005	0.9440
MPV	0.022	0.7640
PLCR	0.037	0.6130
SII	0.138	0.0619
PLR	0.108	0.1430
NLR	0.151	0.0402
BLR	0.045	0.5450
ELR	0.065	0.3800
SIRI	0.064	0.3900
AISI	0.060	0.4160
SIMI	-0.009	0.9000

**Table 3:** Comparison of hematologic biomarkers between clinical response of standard-dosed antihistamine therapy in CSU patients.

Biomarker	Non-responders	Responders	*P* value
MONO	0.37 (0.1)	0.38 (0.14)	0.578
NEUT	4.43 (1.85)	4.1 (1.65)	0.129
PLT	264.85 (51.92)	257.68 (55.08)	0.425
LYM	2.19 (0.59)	2.03 (0.52)	0.128
EOS	0.17 (0.22)	0.14 (0.09)	0.967
BASO	0.02 (0.01)	0.02 (0.02)	0.927
PCT	0.25 (0.05)	0.24 (0.05)	0.206
WBC	6.95 (1.62)	6.55 (1.62)	0.139
PDW	15.87 (1.26)	15.87 (1.26)	0.626
MPV	9.65 (1.1)	9.41 (1.16)	0.228
PLCR	23.93 (7.62)	22.39 (7.86)	0.225
SII	589.91 (348.88)	565.44 (332.39)	0.691
PLR	129.54 (46.23)	135.42 (46.54)	0.323
NLR	2.19 (1.18)	2.19 (1.32)	0.745
BLR	0.01 (0.01)	0.01 (0.01)	0.754
ELR	0.08 (0.08)	0.07 (0.05)	0.608
SIRI	0.81 (0.46)	0.89 (0.89)	0.825
AISI	219 (150.19)	227.09 (207.77)	0.736
SIMI	46.88 (19.14)	50.26 (23.31)	0.644

**Table 4:** Comparison of hematologic biomarkers between clinical response of updosed(fourfold) antihistamine therapy in CSU patients.

Biomarkers	Non-responders	Responders	*P* value
MONO	0.35 (0.15)	0.51 (0.76)	0.814
NEUT	3.83 (1.3)	4.49 (1.52)	0.559
PLT	254.91 (60.57)	271.03 (65.21)	0.682
LYM	2.21 (0.66)	2.12 (0.56)	0.732
EOS	0.14 (0.21)	0.12 (0.08)	0.183
BASO	0.02 (0.08)	0.02 (0.02)	0.911
PCT	0.24 (0.05)	0.26 (0.07)	0.532
WBC	6.46 (1.79)	7.18 (1.74)	0.444
PDW	15.79 (0.46)	15.27 (2.08)	0.235
MPV	9.48 (1.06)	9.35 (0.95)	0.416
PLCR	22.78 (7.45)	21.67 (6.88)	0.324
SII	479.78 (230.98)	620.14 (299.23)	0.598
PLR	123.63 (32.91)	134.56 (41.88)	0.936
NLR	1.86 (0.88)	2.22 (0.81)	0.589
BLR	0.01 (0.04)	0.01 (0.01)	0.851
ELR	0.07 (0.09)	0.06 (0.03)	0.179
SIRI	0.66 (0.68)	1.21 (2.17)	0.805
AISI	167.48 (172.4)	340.45 (614.42)	0.868
SIMI	42.39 (27.22)	72.23 (130.49)	0.883

**Table 5:** Comparison of hematologic biomarkers between with and without allergy history CSU patients.

Biomarkers	Without allergy history	With allergy history	*P* value
MONO	0.37 (0.12)	0.41 (0.15)	0.142
NEUT	4.06 (1.68)	4.34 (1.78)	0.309
PLT	258.28 (56.1)	269.34 (70.79)	0.282
LYM	2.13 (0.58)	2.04 (0.61)	0.569
EOS	0.15 (0.09)	0.14 (0.26)	0.008*
BASO	0.02 (0.01)	0.03 (0.08)	0.150
PCT	0.24 (0.05)	0.26 (0.06)	0.138
WBC	6.69 (1.75)	6.8 (1.73)	0.613
PDW	15.71 (1.54)	15.65 (1.58)	0.965
MPV	9.47 (1.14)	9.57 (1.02)	0.752
PLCR	22.67 (7.88)	23.29 (6.74)	0.646
SII	529.08 (281.9)	676.64 (513.05)	0.385
PLR	128.68 (41.04)	146.61 (67.45)	0.333
NLR	2.04 (1)	2.47 (1.78)	0.325
BLR	0.01 (0.01)	0.02 (0.04)	0.218
ELR	0.07 (0.05)	0.06 (0.08)	0.018*
SIRI	0.78 (0.54)	1.11 (1.22)	0.130
AISI	204.65 (149.01)	296.03 (295.77)	0.178
SIMI	47.54 (21.8)	59.23 (31.39)	0.081

*p <.05.

*Disease Severity*

Among 186 patients with available UAS7 data, only NLR demonstrated a weak positive correlation with disease activity (*r* = 0.151, *p* = 0.040), suggesting that elevated NLR may reflect greater urticarial burden. Other hematological or inflammatory indices, including SII, SIRI, and platelet-derived parameters, showed no meaningful association with UAS7 scores (**Table 2**).

*Treatment Response*

Evaluation of treatment response to standard-dosed antihistamine therapy (74 responders and 74 non-responders after 1:1 matching with 0.2 caliper) demonstrated no statistical differences in any examined parameter (all *p* > 0.05; **Table 3**). Likewise, in the updosed antihistamine therapy subgroup (33 Non-responders and 157 responders; 1:1 matching with 0.2 caliper SMD < 0.1), hematological and inflammatory indices were comparable between groups, with no statistically significant differences (**Tables 3**, **4**).

*Allergic History*

Finally, comparison according to allergic history revealed that patients with a self-reported allergic history (*n* = 30, matched 1:3 with 154 without allergy) had significantly lower eosinophil counts (0.15 ± 0.09*vs*. 0.14 ± 0.26, *p* = 0.014) and decreased ELR (0.06 ± 0.08 *vs*. 0.07 ± 0.05, *p* = 0.018), although the observed differences occurred within the normal reference range for eosinophil counts. Other systemic inflammatory indices (SII, NLR, PLR, BLR, SIRI) did not differ significantly between the two groups (**Table 5**).

Together, these findings indicate that among various systemic immune-inflammation index, NLR weakly reflected CSU activity, while eosinophil-related variations appeared to contribute to differences in allergic background.

## Discussion

In our study of Chinese individuals, we found that patients with CSU exhibited a distinct hematologic profile compared with healthy controls: lower WBC, neutrophil, and lymphocyte counts; higher platelet metrics (MPV, PLCR); lower PDW; and reduced inflammatory indices (SII, SIRI, NLR, AISI). These patterns remained directionally consistent across two sensitivity approaches (1:1 PSM and IPTW). For disease severity, only NLR showed a weak correlation with UAS7 (r = 0.151). No systemic immune-inflammation index differentiated standard-dosed and updosed-antihistamine responders from non-responders. Patients with a self-reported allergy history showed lower eosinophils and lower ELR levels. These findings indicate that complete blood collection based systemic immune-inflammation index capture the inflammatory signature of CSU, thereby addressing an important knowledge gap and establishing a reference for future clinical applications and research.

Neutrophils produce many inflammatory mediators and cytokines and are involved in a variety of systemic autoimmune diseases. Paradoxically, we observed significantly lower neutrophil and lymphocyte counts in CSU patients compared to controls. This may be attributed to two factors. First, the “recruitment hypothesis” suggests that circulating neutrophils and lymphocytes extravasate into skin lesions, aligning with the perivascular infiltration characteristic of CSU pathology. Unlike acute inflammation, where the systemic mobilization of cells typically leads to elevated circulating markers. Second, the non-CSU controls from health examination-based control group may have had subclinical inflammatory states (e.g., metabolic stress) that elevated their baseline counts. Meanwhile, the seemingly counterintuitive finding of lower NLR, SII, SIRI, and AISI in CSU than controls is algebraically consistent: these indices are neutrophil-weighted, and in our CSU cohort neutrophils decreased proportionally more than lymphocytes. While the recruitment hypothesis is biologically plausible, it remains speculative due to these potential baseline confounders. Further studies using paired tissue-blood samples are required to confirm these findings.

For disease severity, only NLR showed a weak positive correlation with UAS7. This paradox may be attributable to disease activity and endotype distribution, which strongly influence leukocyte profiles. Low-activity or type IIb-predominant CSU can exhibit relative lymphocytosis that lowers the NLR below control values (14). This interpretation is consistent with the heterogeneous associations between NLR and disease severity reported across studies. Therefore, we should exercise caution in NLR as a biomarker for disease severity. Its clinical utility remains limited at the individual level, though it may still serve as a cost-effective, supplementary tool for assessing systemic trends in larger-scale epidemiological studies.

With respect to platelet-related indicators, we observed a distinct pattern of enhanced platelet activation characterized by significantly elevated MPV and PLCR alongside reduced PDW. This profile aligns closely with Ertaş et al. and with mechanistic evidence implicating platelet–mast cell crosstalk in CSU (8, 16–18) The increase in MPV and PLCR suggests a predominant circulation of larger, more reactive platelets, which are known to release various pro-inflammatory mediators upon activation. Interestingly, the concurrent reduction in PDW may reflect a characteristic state of persistent and uniform platelet activation, distinguishing CSU from acute inflammatory states where distribution width typically increases.

In treatment response stratification, systemic immune-inflammation index offered limited predictive value. Neither platelet indices nor leukocyte ratios differentiated standard-dosed and updosed antihistamine responders from nonresponders. This is partly consistent with the small prospective study by Kulumbegov et al. (19).

Notably, the allergy-history subgroup had lower eosinophils and a lower ELR. Although CSU is often divided into type I (autoallergic), typically with normal/high IgE and atopy, versus type IIb (autoimmune), often with low IgE and eosinopenia/basopenia, our observation suggests that atopy does not map directly onto the type I endotype; some atopic patients may display a type IIb-like signature with eosinopenia and consequently lower ELR (20–22). In addition to endotypic heterogeneity, increased tissue recruitment of eosinophils, which has been described in CSU and other allergic conditions, may also contribute to lower peripheral counts and reduced ELR, even in individuals with atopic backgrounds (23). Treatment- and timing-related factors common in atopic care, including intranasal/inhaled/systemic corticosteroids and high-dose antihistamines, can also suppress circulating eosinophils and reduce ELR, with additional variability introduced by seasonal and circadian effects (24–27). Therefore, peripheral eosinophils and ELR should be interpreted with consideration of recent medications, inflammatory activity, and sampling context to avoid endotype misclassification.

Our study has several limitations. Its retrospective, single-center design, single-time-point labs for most analyses, and small advanced-therapy subgroups constrain causal inference and predictive power. While non-CSU controls were rigorously screened, occult subclinical inflammation (e.g., periodontal or metabolic disorders) cannot be entirely ruled out, which may introduce potential confounding variables. Nevertheless, this cohort represents a ‘real-world’ population, providing a pragmatic baseline for comparison. In addition, we did not perform formal endotype stratification to distinguish type I CSU from type IIb CSU. Moreover, endotypes can overlap within individuals, complicating categorical assignment. In our cohort, self-reported atopy conflicted with canonical endotype features (e.g., low total IgE with eosinopenia or basopenia suggestive of type IIb), further undermining the necessity of a *post-hoc* classification.

In conclusion, our study demonstrated that CSU patients displayed distinct hematological profiles characterized by enhanced platelet activation (elevated MPV, PLCR) and altered inflammatory balance (reduced SII, SIRI, AISI, NLR). The weak positive correlation between NLR and UAS7 suggests its clinical utility remains limited as a peripheral biomarker for disease activity. Based on our findings, clinicians may incorporate complete blood collection based systemic immune-inflammation index, such as NLR as a cost-effective, supplementary indicator for assessing systemic trends in larger cohorts. However, further longitudinal studies of pre- and post-treatment changes, are needed to validate our findings and emphasize the potential of utilizing laboratory parameters for CSU management.

## Data Availability

The raw data supporting the conclusions of this article will be made available by the authors, without undue reservation.

## Supplementary material

Supplementary Figure 1 A love plot shows standardized mean differences of baseline covariates before and after propensity score matching between CSU patients and healthy controls.

Supplementary Figure 2 Covariate balance before and after 1:1 PSM.

Supplementary Figure 3 Covariate balance before and after IPTW.
