# Supplementary figures and images for: Is it possible to use complete blood collection based systemic inflammatory indices as potential biomarkers for chronic spontaneous urticaria
Source: Front Immunol. 2026 Mar 9;17:1760879. doi: 10.3389/fimmu.2026.1760879 (PMC13006515; doi:10.3389/fimmu.2026.1760879)

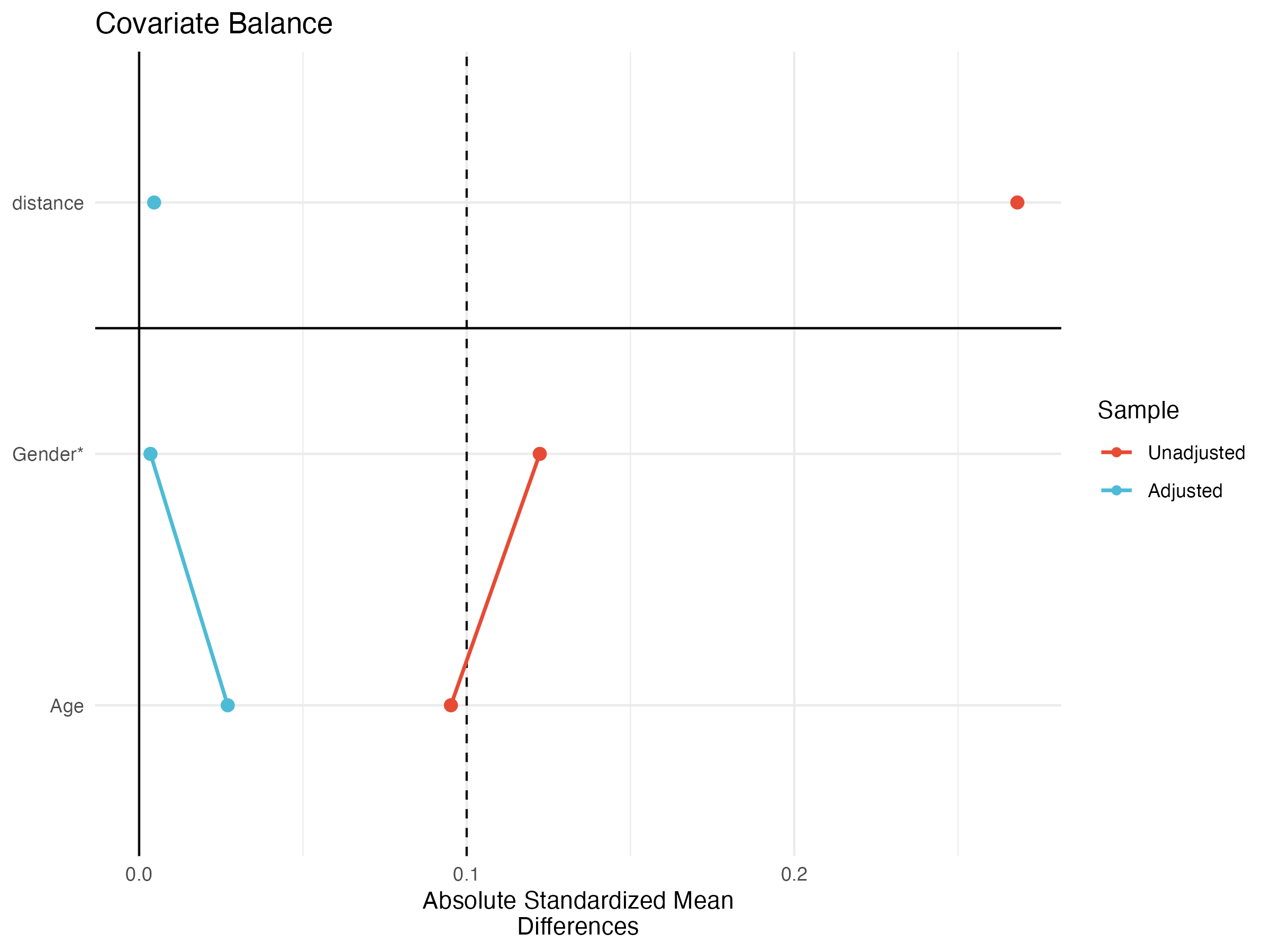

Supplement: Supplementary Figure 1 — A love plot shows standardized mean differences of baseline covariates before and after propensity score matching between CSU patients and healthy controls. [file Image1.tiff]

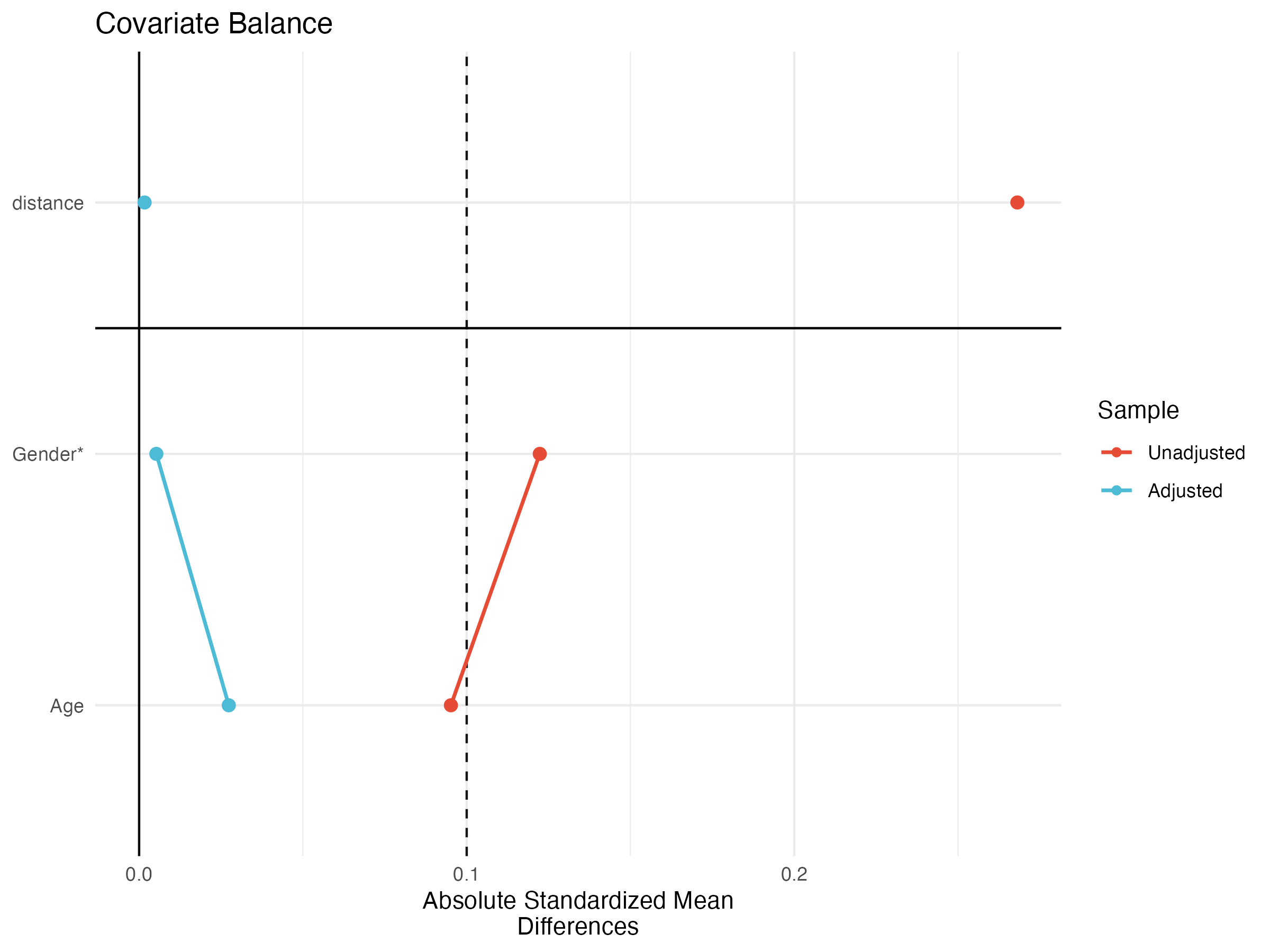

Supplement: Supplementary Figure 2 — Covariate balance before and after 1:1 PSM. [file Image2.tiff]

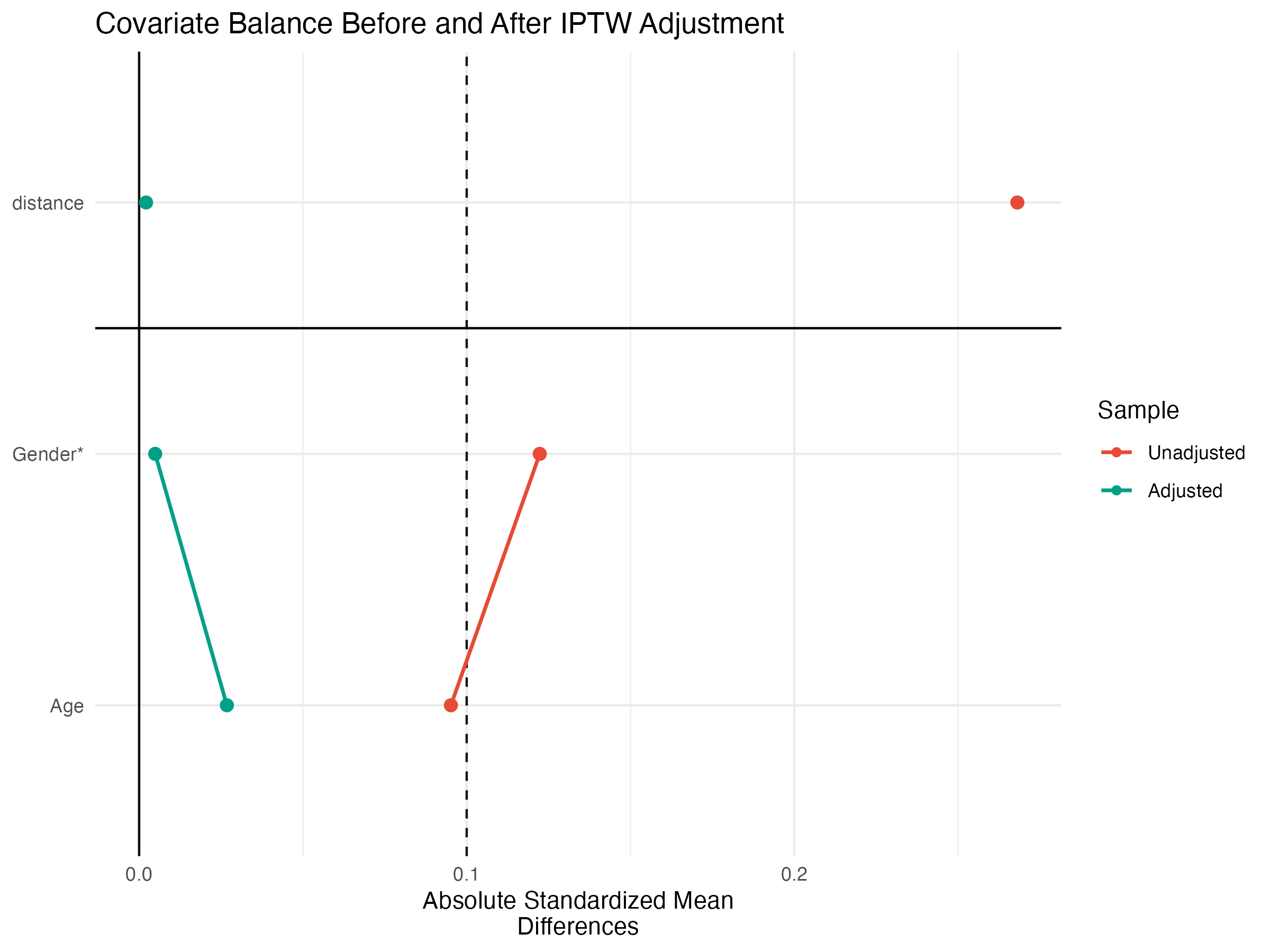

Supplement: Supplementary Figure 3 — Covariate balance before and after IPTW. [file Image3.tiff]
